# Accounting for the Moral Significance of Technology: Revisiting the Case of Non-Medical Sex Selection

**DOI:** 10.1007/s11673-018-9891-4

**Published:** 2018-12-27

**Authors:** Olya Kudina

**Affiliations:** Philosophy Department, University of Twente, Cubicus C315, Drienerlolaan 5, 7500AE, Enschede, The Netherlands

**Keywords:** Sex selection, Technological mediation, Moral mediation, Ethics of technology

## Abstract

This article explores the moral significance of technology, reviewing a microfluidic chip for sperm sorting and its use for non-medical sex selection. I explore how a specific material setting of this new iteration of pre-pregnancy sex selection technology—with a promised low cost, non-invasive nature and possibility to use at home—fosters new and exacerbates existing ethical concerns. I compare this new technology with the existing sex selection methods of sperm sorting and Prenatal Genetic Diagnosis. Current ethical and political debates on emerging technologies predominantly focus on the quantifiable risk-and-benefit logic that invites an unequivocal “either-or” decision on their future and misses the contextual ethical impact of technology. The article aims to deepen the discussion on sex selection and supplement it with the analysis of the new technology’s ethical potential to alter human practices, perceptions and the evaluative concepts with which we approach it. I suggest that the technological mediation approach (Verbeek, 2005, 2011) can be useful to ethically contextualize technologies and highlight the value of such considerations for the informed deliberation regarding their use, design and governance.

## Introduction

The possibility to select a sex of one’s future child prior to pregnancy is not new, with sperm sorting and Prenatal Genetic Diagnosis (PGD), accompanied by In Vitro Fertilization (IVF) representing present-day options. However, ethical concerns accompanied sex selection early on, ranging from worldwide population imbalances and gender discrimination to fear of reinstating eugenic movements. Consequently, non-medical sex selection technology (hereafter SST) faces strict regulation globally, unless medically justified to prevent vertical transfer of genetic diseases; or unless the national law endorses family balancing as a qualifying non-medical motivation. Currently, SST is expensive, invasive and often requires several attempts either in view of the inaccuracy in sperm sorting, or the exigencies of the IVF process. However, a new iteration of SST, in a form of a microfluidic chip for sperm sorting, suggests that a cheap, non-invasive and secure way of sex selection prior to pregnancy could also be possible, and even in the setting of one’s home (MESA+ 2017). This article inquires how such a material change in the sex selection practice can offer new ethical insights and deepen the existing debate regarding non-medical sex selection.

Albeit the ethical scholarship regarding SST is abundant, it largely downplays the role of technology in this context. Several authors have written on the non-neutrality of technology, considering the way technology embeds values, inspiring human actions and understandings (Parens 2015); how it fosters moral engagement and relations (Turkle 2007); and how technology can provide moral insights for people (Wallach and Allen 2009).

This is not to say that technology defines the moral concerns and the ethical norms to approach them. Rather, by virtue of its design, foregrounding some options and disclosing others, technology co-shapes perceptions and actions of people (Ihde 1990; Verbeek 2005). Telecare technologies enable physicians to perceive and treat their patients regardless of geographical proximity, fostering new configurations of moral engagement and responsibility. Medical imaging technologies guide physician’s interpretation of a patient’s health, as well as the way patients perceive and “feel” themselves. The ethical implication of this human-technology intertwinement is that technologies also help to shape moral evaluations and decisions of people, whereby ethical frameworks with which we approach technologies co-evolve with these same technologies (Verbeek 2008, 2011; Kudina and Verbeek 2018). Looking at the new sex selection technology from this angle may provide new and contextualize existing moral concerns regarding its non-medical application, affecting society in ways that often bypass the radar of the policymakers in view of their subtle qualitative nature.

Current deliberations on technological future lack such a contextual sensitivity and give prevalence to the quantification approach of risks and harms. Numerical appraisal of health, safety, autonomy and other values provides a constructive way to deal with these concerns in relation to new technologies, helping “to transform fuzzy, unmanageable ‘hazards’ into specific ‘risks’ that could serve as the basis for policy decisions” (Swierstra 2015, 6). However, such a “hard impacts” logic misses an equal potential of technology to mediate and co-shape the perceptions, practices, responsibilities and value frameworks of people (Verbeek 2011). Accounting for the subtle qualitative impacts of technologies does not invite for a yes-or-no answer regarding the future of a given technology because such impacts “do not fit well within a techno-scientific discourse,” and as such, “they are easily dismissed as romantic, irrational, subjective or vague” (Haen 2015, 21). Yet, the fact that they are difficult to trace does not make them less important.

This article will present an angle at the sex selection discussion, sensitive to the material changes the new sex selection technology (hereafter SST+^1^) suggests and to the sociocultural background against which such a change may occur. As such, it does not intend to pass a judgment on whether it is ethically justified to use this technology. To understand the way SST+ can influence the quality and structure of human practices and experiences, I will rely on the philosophical approach of technological mediation (Verbeek 2005, 2011), which suggests that technologies are not neutral but actively mediate relations between people and the world. The article aims to deepen the discussion on non-medical sex selection, supplement it with the arguments related to the moral significance of technology and highlight the value of such considerations for the informed deliberation on emerging technologies.

The discussion proceeds as follows. I will first provide a technological background and explain how SST+ is different from the current technologies. I will then briefly review the ethical debate regarding SST and position the new technology in it. Next will follow an elaboration on the moral significance of technology, as well as how to account for it in the case of emerging technologies, such as SST+. I will then anticipate and explore the moral implications of SST+ with view to the current SST practice and public deliberation. The article will conclude with the reflections on the moral significance of technology and argue for the necessity of such considerations at any level of decision-making regarding technology’s use and legal standing.

## Technological Background

Assisted reproduction in the form of sex selection became available in the early ʼ90s. Currently, SST is possible via sperm sorting and pre-implantation genetic diagnosis (PGD) (Parliamentary Office of Science and Technology 2003). In sperm sorting, two dominant methods are available: MicroSort, whereby fluorescent dye enables distinguishing X- and Y-chromosomes in vitro; and the Ericsson method, which relies on the higher mass factor of X-chromosome to sort the sperm when it passes through a protein of serum albumin. Sperm sorting itself does not amount to pregnancy, and the accompanying procedures of intrauterine insemination, in vitro fertilization (IVF), intracytoplasmic sperm injection or self-insemination are necessary. PGD requires extraction of female oocytes for an IVF procedure. Once the embryo develops eights cells, one cell is removed for chromosomal DNA analysis, which can reveal the sex of the embryo, among other characteristics (Harper and SenGupta 2012). PGD dominates the market of sex selection because it provides higher selection accuracy than sperm sorting (99 per cent vs. 75-85 per cent) (GenderSelect 2017). However, because PGD necessitates an accompaniment of IVF and sperm sorting has a low selection accuracy as compared to PGD, successful pregnancy depends on additional factors and often requires several attempts. The overall cost of sex selection is high, between US$1,300-3,400 per attempt for sperm sorting; and US$18,000-25,000 per attempt via PGD/IVF (ibid.).

Sex selection faces strict regulation worldwide. However, prevention of the vertical transfer of genetic disorders, such as hemophilia, Lesch-Nyhan syndrome, Duchenne-Becker muscular dystrophy and Hunter syndrome justify the medical use of SST (World Health Organization 2011). A non-medical exception concerns the desired variety of sexes in the family, when parents already have at least one child and want to have a child of the opposite sex. Such “family balancing” application of SST is available in the USA, Cyprus, Ukraine, Israel and several other countries (Bayefsky and Jennings 2015, 54-55). The technology behind sex selection is invasive (particularly with PGD/IVF), expensive and requires multiple rounds of sorting or rounds of IVF. Despite this, non-medical sex selection is one of the top motivations for cross-border medical tourism (Van Hoof, Pennings and De Sutter 2015); and accounts for up to 9 per cent of the PGD/IVF cycles in the US as of 2005 (Baruch, Kaufman and Hudson 2008).

A recent technological development could overcome the above-mentioned constraints (MESA+ 2017; Valkenberg 2014). A microfluidic chip-based technology can measure sperm characteristics, such as motility and morphology, to ameliorate problems with selection of spermatozoa, common to assisted reproductive technologies (ART). However, an adapted version of the technology could also be used for sex selection since apart from cell viability and acrosome state, the microfluidic chip can also analyze the chromosomal content of individual sperm cells (De Wagenaar et al. 2015, 1295). It would follow similar logic as the existing sperm sorting techniques: the fact that X-chromosomes are longer and therefore heavier than Y-chromosomes. The microfluidic chip operates at nanolevel, where the chromosomal variation in weight would be significant to determine the chromosomal sex, making sex selection in principle possible. One would need to present a sperm sample on a chip, which would sort the sperm in X- and Y-bearing groups, providing two options depending on the desired outcome. A chip-based form of sex selection, in view of its non-invasive nature, offers home setting as a potential application site. Form-wise, it would likely take after its predecessor, an at-home microfluidic chip for the assessment of semen quality, whereby a disposable chip, requiring small volumes of sperm sample, would be “used in combination with a handheld measurement system and management software” (Segerink et al. 2012, 66). Moreover, at-home application would significantly reduce the costs of sex selection and bring it outside of the medicalized domain.

It is important to underline that the confidentiality of the research and its still predominantly open developmental avenues permit limited view on the technical specifications of SST+. Although researchers confirm the successful proof of concept of certain sperm characteristics (De Wagenaar et al. 2015), there is no word of pursuing the sex selection trajectory for human use. It is not clear how SST+ will manage the cells with an extra or missing chromosome (a chromosomal aneuploidy), although researchers previously used the on-chip analysis using staining protocols to successfully test for chromosomal anomalies in individual sperm cells (De Wagenaar et al. 2015). On a practical level, carrying out the whole sex selection process at home implies not only the point-of-care sperm sorting, promised by SST+, but also self-insemination, akin to using a turkey baster or a disposable syringe for at-home insemination using donated sperm. Self-insemination at home presents limitations and often necessitates multiple attempts to lead to pregnancy (Wikler and Wikler 1991). Although the form and usability of the technology remain unknown, anticipations of a chip “for 12,95 at the drugstore” (Valkenberg 2014, ¶16) suggest the expected resemblance to a pregnancy test in terms of cost, ease of use and acceptability. Some anticipate that “The [sex] selection chip […] has the potential to become available at a large scale and a low price; therefore, the social effect is likely to be quite substantial if the technology would indeed be introduced” (Verbeek 2015, 197).

Importantly, as mentioned earlier, sex selection for non-medical reasons remains illegal in many countries, with a rare exception for family balancing. At the same time, history suggests close intertwinement of technological innovation, public views, moral frameworks and legislation. SST originally appeared to increase the birth of female animals to boost agricultural outputs, but later migrated to use in humans. Both the research and the history of SST indicate no technical obstacles to using SST+ for people and that it is in principle possible (Valkenberg 2014).

In what comes next, I would like to consider the potential leap of SST+ to human use, although I want to stress that there is no support for pursuing this agenda from the technology developers. Nonetheless, such an anticipative exploratory study is useful and necessary, as a testbed for considering previously unacknowledged moral significance of the material setting in the sex selection practice. Moreover, in the hypothetical event of market introduction, this study can offer matters of concern for a responsible design and governance of SST+, a technology that, as witnessed above, still has an open future regarding its form, usability and societal embedding.

In the present study, the physical or market presence of SST+ is not essential (albeit desirable) for identifying its potential moral significance. Sex selection technology already permeates the minds of people in the form of technological visions, promises, concerns, fears and hopes, all of which mediate technological appropriation not only in theory, but also in practice (Grunwald 2016). As Boenink, Swierstra and Stemerding (2010) showed, the technological promises and visions mobilize societal and scholarly fears and hopes, which can be instructive for understanding the moral significance of a technology yet to materialize. Although an empirical investigation would substantiate the study, an anticipative exploration could still be plausible by grounding in practices with the similar existing technology and informed by the societal and ethical discourse (Lucivero 2012). In short, an inquiry into technological potential to mediate public (moral) views, routines, and habits can be informative in the wake of a technology potentially at society’s doorstep, as is the case of SST+.

Ethical considerations regarding the societal risks of SST are what ultimately informed its strict regulation for non-medical reasons. The next section will briefly review the debate to date and position SST+ within the dominant arguments to inquire how the new technology faces the old challenges and alters the debate, if at all.

## Understanding the Ethical Debate Regarding SST

The risk-benefit considerations polarize the discussion concerning sex selection in two camps: those who consider that the possible harms of this technology render it impermissible and those who suggest that the possible benefits of non-medical SST outweigh potential risks.

Those who endorse sex selection suggest that people should be free to make reproductive choices without the guidelines of the government or anyone else, insofar as these choices do not limit the freedoms of others (Dickens 2002; Savulescu 1999). Some consider SST beneficial for reducing abortion rates and diminishing the suffering of women, giving birth to undesired children; and children, who would live the life knowing they were unwanted (Puri and Nachtigall 2010). Others suggest that SST empowers women and can decrease violence towards them (Steinbock 2002). Thus, the proponents of non-medical SST justify their case by invoking the procreative rights and liberties, as well as alluding to the best interest of a mother and a child.

The worry of catering to preferences of prospective parents and disregarding the inherent worth of a child marks the case against sex selection (Sandel 2004). Some suggest that SST will start a race to “designer babies” and enact the slippery slope of eugenics (Strong 2001; Smith and Taylor-Sands 2018). Others worry that SST is discriminative by design, particularly with PGD/IVF, when “a single trait […] obliterates the whole” (Parens and Asch 1999, 13). Following this thought, SST for family balancing is also impermissible because it reinforces heteronormativity, fosters medicalization and misinterprets reproductive autonomy of parents (Shahvisi 2018). SST transforms sex into a paramount trait when considering a future of an embryo (Wertz and Fletcher 1992). Ascertaining the sex of an embryo becomes sufficient to either allow its gestation or discard it altogether. This invites the questions regarding the moral status of an embryo relative to it potentially developing into an autonomous child with independent moral standing; and in parallel, the questions of management strategies regarding the surplus embryos (ibid.). Moreover, Browne (2017) and Hendl (2017) argue that because SST frames the sexes as not interchangeable, it promotes sexism, fosters unreasonable gender expectations in the prospective parents and reduces their reproductive autonomy.

Those who oppose SST predominantly assume global preference towards selecting males. There is a worry that SST could provoke worldwide sex-ratio imbalances, challenging the sociocultural standing of women and even diminishing it to their childbearing abilities (Hvistendahl 2011). However, many argue that because the sex/gender preference empirically relates to the dominant sociocultural views, it is not a generalizable assumption and should not be the primary rationale for banning SST (cf. Dahl et al. 2003; Gleicher and Barad 2007).

The ethical debate on SST indicates strong polarization into dominant groups of proponents and opponents of the technology, essentially pondering whether sex selection should be allowed. Several policies and regulations reflect this ethical debate, suggesting that the risks of the potential harms to the children and potential population imbalances outweigh the potential benefits to the prospective parents, rendering the use of non-medical SST impermissible (Human Fertilisation and Embryology Authority 2002; European Parliament 2013).

## Positioning SST+ in the Existing Debate

The debate around SST encompasses diverse ethical concerns and anticipated benefits. SST+ suggests a close-to-certain and safe selection outcome at low cost and with home use, which would also eliminate the need for creating and discarding the embryos through PGD. I will now inquire whether such a change in the material setting of the sex selection practice addresses any of the ethical concerns outlined above.

One may suggest that for prospective parents, SST+ promotes the four bioethical principles (Beauchamp and Childress 2001) and provides a new set of arguments to support sex selection. SST+ could be beneficent to prospective parents by allowing to independently pursue the desired reproductive agendas. It could similarly support the principle of autonomy^2^. SST+ could also foster the principle of non-maleficence to a greater degree than the currently existing sex selection methods because it is non-invasive and obliterates the involvement of embryos. Finally, SST+ could be just by allowing equitable access to the technology at low cost and ease of use.

However beneficial SST+ could be for the prospective parents, in relation to the future children the ethical concerns remain intact. Even though SST+ could bypass the ethical concerns regarding embryo creation and management, it would not change the potential to promote sexist attitude and psychological risks to a child.

Therefore, SST+ could ameliorate some ethical concerns but leave others intact. In parallel, SST+ could foster novel ethical challenges, if only due to the promise of being more affordable and non-invasive. By significantly altering the material setting of sex selection, it can affect society in ethically nuanced, subtle ways that could gradually inflict social and moral change in individual and public views. In other words, SST+ could mediate the moral landscape of human beliefs and practices in ways different from the existing sex selection methods.

## Considering the Mediating Essence of Technology

Because technologies help to co-shape what counts as real, possible and reasonable, they are not neutral. The technological mediation approach explores how technologies shape relations between people and the world around them (Ihde 1990; Verbeek 2005). When in use, technologies are far from neutral “objects” in the hands of autonomous human “subjects;” rather, they form a medium that enables a distinct representation of the world and mediate the way people relate to themselves and the world around (Rosenberger and Verbeek 2015).

Consider how an ultrasound in the prenatal care co-shapes the care relationship between the prospective parents and their unborn child (Verbeek 2008). The parents see the unborn through the screen that portrays the child as detached from the body of the mother. The visualizations on the screen also allow the unborn to be gendered and framed as a patient, in view of possible genetic conditions the ultrasound may reveal. The parents might not want to know whether their future child has a genetic condition. However, they know that the ultrasound image can reveal it, which makes it difficult to avoid the ethical questions of further care for the child, with abortion being one of the options. By framing the unborn in a certain way, the ultrasound technology mediates the care relations between the child and the prospective parents, enabling certain perceptions of the child and the parents, as well as various practical manifestations of the value of care.

The ultrasound example also illustrates the ethical implications of the technological mediation approach: “If ethics is about the question of ‘how to act’ and ‘how to live’, and technologies help to shape our actions and the ways we live our lives, then technologies are ‘actively’ taking part in ethics” (Kudina and Verbeek 2018, 7). Technologies mediate the moral inclinations, habits, norms and values of people by co-shaping their moral perceptions and actions: ultrasound technology mediates moral questions about procreative rights and liberties; telecare technologies mediate moral experiences of physicians; and the contraception pill enabled revision of the moral views on sexuality and the valuations of homosexuality, having loosened the link between sex and reproduction (Mol 1997, 8). That technologies mediate morality does not imply any form of moral agency in technologies. Rather, what the examples above and the technological mediation approach suggest is that moral agency does not pertain solely to either people or technologies, but rather to their hybrid entanglement (Verbeek 2011).

Just as any ethics-related aspect, the moral significance of technologies is difficult to identify, anticipate and manage. More so, when a technology in question is still under development. Nonetheless, an encompassing and informed decision-making on emerging technologies would be incomplete without accounting for their qualitative ethical implications. The approach of technological mediation offers one way to accomplish this task. It suggests that the locus of morality lies in the interaction of people and technologies (Verbeek 2011). Therefore, to explore and analyze the ways in which technologies co-shape, enable, challenge or change in any way the (moral) engagement of people with the world, one has to scrutinize the practices that technology enacts or destabilizes (Mol 2002). SST+ would enact a different material context for the practice of sex selection from the one currently available via PGD/IVF and sperm sorting. In the following section, I use the technological mediation approach to anticipate and understand the possible societal impact of such a material change in sex selection in the context where conceptual appropriation of SST+ is already possible, owing to the promises, concerns, hopes and fears that surround its development.

All discussions about the future of technologies carry a note of speculation. With SST+, the few known features prompt new and build on the existing ethical questions about sex selection. When anticipating the mediating effects of SST+, I try to avoid speculation by grounding in the philosophical approach of technological mediation, literature studies and careful assessment of technological visions regarding SST+. I also consider retrospective and current examples of biotechnologies in society. Such examples provide a sense of where and how possible tensions in practices and values may arise, bearing in mind the non-linearity of technological development. As such, I invite the readers to view the following mediation analysis of the potential ethical implications of SST+ as explorative, non-exhaustive and anticipative, providing a springboard for further critical discussions and empirical exploration.

## Exploring the Mediating Effects of SST+

The potential ethical implications of SST+ stem from the anticipated changes, broadly classified in three clusters, namely: the demedicalization of the sex selection practice, the transformation of SST to a direct-to-consumer technology and the move of SST to an open market. The clusters represent overarching themes across specific ethical considerations, identified through literature analysis. The themes often overlap and certain ethical concerns within them could fit multiple categories. The purpose of the cluster scheme below is to allow a somewhat structured navigation of the ways in which SST+ can mediate the moral landscape.

1. Demedicalization of the Sex Selection Practice

Shahvisi (2018, 135) suggests that “The authority and infrastructure of healthcare should not be used to facilitate practices [of SST] which play upon misunderstandings and perpetual injustice.” Yet what happens when SST withdraws from the medical domain? A major difference of SST+ in relation to the existing techniques is the potential move from the clinical to the home setting. Currently, sex selection predominantly implies PGD/IVF, which requires time, financial investment, logistical planning, invasive physical interventions and often results in psychological burden to the prospective parents (Franklin and Roberts 2006). SST+ could *demedicalize the practice of sex selection* by offering an affordable, non-invasive technique for use at home.

Performing sex selection at home can *reintroduce elements of the value of naturalness* in pregnancy-related practices, facilitated by technology. It can also enhance the value of privacy in the family planning practice, regarding not only the privacy of decision-making, but also concerning the bodily privacy, by allowing prospective parents to have the procedure at home, without an involvement of the third party. As such, SST+ could facilitate the value of naturalness in assisted reproduction and enhance the procreative liberties and autonomy of the prospective parents.

Interestingly, SST+ could *increase the role and importance of men in the reproductive process*. Some argue that ART increased the autonomy and procreative rights of the prospective mother and diminished the significance of a father in parenting, contributing to “the end of men” (Rosin 2012). SST+, on the contrary, could highlight the significance of the prospective father by giving him a primary role in the beginning of the conception journey. SST+ requires proactive decision-making from a father-to-be by asking whether he wants to determine the sex of his future child (hopefully in discussion with a prospective mother); and if so, demands an active contribution. Sexing of a child is one of the key practices in transition to parenthood and attaining the moral status and responsibility of parents (Schadler 2014). By placing the sexing process ahead of obtaining evidence about pregnancy and the health status of the embryo, SST+ allows prospective parents to transition to parenthood and assume parental roles and responsibilities much earlier. By placing the prospective father in charge of the sexing process, SST+ uplifts his role and importance in decision-making on having children. The increase in paternal role can mediate the parental relations and power balance in different ways, from levelling the responsibilities and participation in childbearing to fostering patriarchal authority and exploiting the power of required male role with SST+.

2. SST+ as a Direct-to-Consumer Technology

Framing SST+ as an on-demand direct-to-consumer technology assumes the status of *prospective parents as customers*, not as patients, continuing the trend started by ART. SST+ as an open market technology, directly available to prospective parents without medical oversight, could further exacerbate the shift from patients to customers, as well as reaffirm the commodification of reproduction. SST+ would nearly eliminate the need for medical assistance, in parallel offering the pre-pregnancy sexing as a new norm in reproduction, available at a local drugstore at low cost. SST+ appears here as a facilitator to realize parental choices. The history of PGD indicates that assuming a customer status provides an enhanced feeling of entitlement to choice and increases the perception of control over one’s destiny (Franklin and Roberts 2006). Such an increased sense of autonomy with the patient-to-customer shift in SST+ could mediate parental perceptions and expectations of themselves and their child.

SST+ as a direct-to-consumer technology could further intensify an existing *fate vs. choice discussion* in assisted reproduction. In order to determine the moral status of sex selection practice, its opponents often appeal to parental virtues (Sandel 2004; McDougall 2005). Assuming that unpredictability is one of the facts of nature, to enable a child’s flourishing, a good parent needs to possess a virtue of acceptance. Following this logic, SST+ would entice a parent to perform a wrong parental action because selecting a sex of a child means violating the virtue of acceptance. Hence, SST+ would be morally wrong. Yet if we look at the technology through the lens of mediation approach, we seem to take for granted an assumption that “unpredictability […] is an intrinsic feature of human reproduction” (McDougall 2005, 603). Merely an existence of SST challenges that assumption. The sex of a baby is now available for choice. Whether a couple chooses to select a sex of their child or not, is up to them, but it has escaped the domain of fate and is now a profoundly technologically mediated option. By significantly simplifying the process of sex selection, SST+ can decrease the factor of unpredictability in childbearing, creating a threshold to the unconditional acceptance, traditionally associated with good parenthood.

Transforming SST into a direct-to-consumer low cost and at-home technology may invite public curiosity, feed into the existing interest on sex selection and facilitate acceptability of the practice. Moreover, a change in the material setting of SST+ may invite prospective parents who never considered the possibility of sex selection to do so. After all, if the technology promises to be affordable, safe and easy to use, it could invite the prospective parents to answer the question *why not* to use SST+. This may provide additional stress and anxiety in justifying private reproductive choices. Therefore, while SST+ may increase parental autonomy and reproductive liberty, the “Why not?” reconfiguration may simultaneously decrease them, creating a sense of pressure to justify private choices.

3. The Move of the SST+ to an Open Market

The transfer of SST from the medical to the open market domain can affect the way people approach the subject of *responsibility regarding sex selection*. The questions of responsibility related to SST+ are multidimensional and involve different actors. Judging from historical and current cases of ART, the concerned stakeholders in the case of SST+ include at least prospective parents, the industry developing this technology and bringing it to the market, and medical professionals.

One could argue that the “Why not?” attitude that SST+ could facilitate denigrates the responsibility of *prospective parents* by commodifying children and inciting consumerism in the childbearing decisions. I would argue that SST+ mediates the value of responsibility by simultaneously bringing its multiple dimensions to the fore: while it may invite a simplified perception of complex decision-making regarding the childbearing (as suggested above), SST+ also brings about a new responsibility to recognize the inherent uncertainty that accompanies parental choices regarding sex selection. Prospective parents routinely face responsibility for their reproductive choices, when choosing to conceive a child or facing an unwanted pregnancy, deliberating on how to manage pregnancy, how to raise a child, etc. However, SST+ confronts prospective parents with an additional responsibility, the one that comes with using the technology.

Prospective parents might rejoice at the opportunity to fulfil their preferences and, some may say, a natural right to decide on the future of their children. While SST+ highlights the option to choose, the approach of technological mediation helps to reveal what the technology foreshadows. If one frames SST+ as a manifestation of parental rights and liberties, as the right to choose, this would also entail a duty, a responsibility to accept the limitations of that choice. While SST+ allows parents to select the biological characteristics typical to one sex or the other, it does not change the inherent uncertainty regarding the sociocultural inclinations, identifications, and preferences of the child. The way prospective parents take up this responsibility depends on their personal histories and dynamic sociocultural contexts, which may result in multiple scenarios of parent–child relations depending on the degree to which parents enforce their perceptions and expectations on the child. In parallel, public discussion forums, medical practitioners, and government Technology Assessment efforts can help prospective parents to make informed decisions about the use/non-use of SST+ and its consequences by helping to scrutinize the technological promises and to recognize and account for the ethical implications of this technology.

*The industry and particularly its marketing branches* have a responsibility to ensure that prospective SST+ users make informed choices about the use (or non-use) of their product. Marketing informs the mindset and facilitates the decisions of prospective customers, thus influencing the way people appropriate the object of advertisement. SST+ is not a neutral diagnostic tool but a technological mediator that contributes to shaping the choices of people about the future of their offspring. With regard to SST+, it is important to deliver a balanced message, emotionally and scientifically, about what SST+ can and cannot do. For instance, it would be misleading to foster a connection in the minds of people between choosing the sex of the sperm and having a successful pregnancy; or about choosing the biological sex of a child and the gender identity they develop later in life. The complicated relationship between sex and gender has received a considerable amount of scholarly attention (c.f. Goffman 1979; Eckert 2017). In particular, Goffman (1979) has exposed and analysed the influence of the mass media and advertising on the sex/gender discussion and public perception of gender roles and identities. SST+, with only two options to choose from and the possible “Why not?” attitude, can further challenge the sex–gender discussion in society. Promoting SST+ with direct appeal to preferred gender characteristics could further inscribe public stereotypes about the direct correlation of sex and gender and reduce the visibility of other possible gender identities.

The stakeholders who develop technology and put it to market always have responsibility towards their consumers and the public at large, at least regarding the safety, security, and other “hard impacts” regarding the direct use of their product. As history shows, an open market setting under a legislative regime that cannot keep up with the pace of technological development offers little incentive to private companies for ethical self-reflection beyond the legally-mandated requirements. However, recent developments in the direct-to-consumer genetic testing business indicate that such efforts can arise within the industry itself in an effort to innovate responsibly and address ethical concerns. Addressing the consumer boom with genetic testing technologies, private genetic testing companies collaborated with a public think-tank, including academics, legal practitioners, and advocacy groups, to produce self-imposed industry-wide ethical guidelines (Future of privacy forum 2018). The impact and effectiveness of these guidelines have yet to be ascertained but, “While no policy could ever eradicate risks for consumers who agreed to give their DNA, the industry guidelines are attempting to address some of the biggest fears” (Brown 2018, ¶25). However impactful these guidelines might be, they indicate an acknowledgment on behalf of the industry of their responsibilities beyond the direct use of their product and offer an example for other direct-to-consumer diagnostic tools, such as SST+.

Finally, it is easy to assume that if SST+ moves from a medical to a private domain, it would eliminate the involvement of the medical professionals. Drawing again on the example of direct-to-consumer genetic testing, the transition may not obliterate the role and responsibility of medical professionals (Lovett Rockwell 2017). Lack of market oversight and accountability may exploit the hopes and fears of prospective parents, leading them back to the doctor’s office to help navigate the complexities of the selection and conception process (emotionally and practically). This could bring medical context back into the equation, raising questions regarding physicians’ responsibilities and burden on the scarce resources when the original cause is not medically justified.

## Discussion

Overall, moral mediation analysis makes visible how the new material setup of SST+ could enable a new set of sex selection practices, change their nature, foster new ways of perceiving sex selection from individual and societal points of view, and crystalize new power relations in the process of childbearing.

Firstly, the move of sex selection to the setting of one’s home could demedicalize and normalize the practice of sex selection. With this, SST+ could reinforce the values of naturalness and privacy for its users. In parallel, SST+ could highlight the role of men in the reproductive process. This could mediate parental relations in multiple ways, from levelling the power balance in childbearing to abusing the newly acquired patriarchal authority.

Secondly, making SST+ an affordable direct-to-consumer technology could mediate the self-perception of its potential users. On the one hand, it could intensify a shift from patients to customers, entitled to the right of choice. On the other hand, from parents being subject to fate in childbearing to parents actively determining it. Such shifts could co-shape new moral understandings of what good parenthood means. In parallel, significantly simplifying the process of sex selection and reducing its price could foster a societal “Why not?” attitude, making private reproductive choices available for reflection and potential justification.

Thirdly, the potential move of SST+ to an open market could mediate the value of responsibility in relation to different stakeholders. Introduction of SST+ could foster different manifestations of parental responsibility in the course of evolving personal histories and sociocultural contexts; contextualize industry and marketing responsibilities towards ethical reflection beyond immediate use of the technology; and maintain the role and responsibility of medical professionals in figuring out the multiple nuances that could materialize during the use of SST+.

The technologically mediated ethical concerns outlined above do not represent an exhaustive list but rather scratch the surface of possible moral dynamics based on the limited knowledge we have about SST+. But however preliminary, technological mediation analysis explores moral contingencies on both individual and societal levels concerning the balance of parental roles, the sex–gender relationship, responsibility, and choice. Most importantly, it highlights the potential of SST+ to mediate the moral landscape and the pervasiveness of its implications. With a mediation approach, SST+ appears morally contextualized and substantiated, lending additional and different information for deliberation and informed decisions regarding it.

We live in an age when SST is a viable option that people seek to obtain notwithstanding cost, safety, international borders, and regulation. SST+ suggests material change in sex selection, potentially rendering it affordable, non-invasive, and home-based. Whether or not this technology materializes, the moral dynamism that this article exposes in relation to the still nascent SST+ suggests considering the moral charge of technologies early on, rather than waiting for their introduction. Moreover, it suggests an expansion beyond the existing risk-and-benefit logic and the quantification of potential harms to also explore the way technologies mediate values and normative standards. Such an expansion, both on the levels of ethics and policymaking, is necessary to understand how SST+ could reorganize moral and political frameworks of action, and to reflect on how to deal with it in an informed way.

Expanding ethical reflection on sex selection along these lines would also help to account for local cultural contexts and beliefs. The anticipated psychological harms and procreative liberties of SST already manifest differently in different sociocultural contexts. The ethical implications of SST+ will also materialize in interaction with local environments. In this light, an informed reflective engagement with SST+ is possible only with the situated, context-dependent understanding of its moral mediation potential.

Incorporating the intuitions of the technological mediation approach in the deliberations over a future of technologies in question would also help to account for the dynamic nature of the ethical concepts with which we approach these same technologies. As suggested by the analysis above, the possibility of sex selection mediates the virtue of good parenthood by lowering the degree of acceptability towards the uncertainty, traditionally ascribed to virtuous parents. Approaching technologies with static pre-given concepts of moral character would thus miss the scope of their mediating potential. As illustrated by the case of SST+, the technological mediation approach can contextually substantiate the values and conflicts at stake, as well as illustrate their provisional and dynamic nature.

Expanding ethical reflection on technologies along the lines of moral mediation further complicates the process of ethical analysis. However, it indicates a principle change in reasoning about technologies and the necessary moral expansion of the reflective stance toward them. Ensuring informed engagement with technological mediations necessitates a reflective stance toward the way technologies co-shape the context of our everyday existence and co-organize moral infrastructure for our decisions. To substantiate the anticipated mediations of SST+ requires additional knowledge about the new technology. It also invites further empirical research, incorporating potential users and non-users, as well as a reflection of their rationale for using or refraining from using this technology. This article presents a starting point for further research and invites ethical consideration of the moral significance of technologies early on rather than waiting for their market introduction.

## Footnotes

1 Please, note that “SST+” denotes potentially expanded technological features of the microfluidic chip for sperm sorting as compared to traditional sperm sorting and PGD/IVF. This does not imply a better sex selection technology, either in practical or moral sense.

2 One could object here with view to the assumption in SST regarding the fundamental differences between male and female sexes. By this token, according to Browne (2017, 196), any SST would reduce the reproductive autonomy of the prospective parents because: “1. It clouds parent’s perceptions of what is possible in parenting, and 2. It entrenches the current social pressure to raise the sexes in very different ways.” As such, one could also consider SST+ maleficent and reducing the autonomy of the prospective parents.
